# The effect of tuina on ulcerative colitis model mice analyzed by gut microbiota and proteomics

**DOI:** 10.3389/fmicb.2022.976239

**Published:** 2022-11-29

**Authors:** Hourong Wang, Zhifeng Liu, Tianyuan Yu, Yingqi Zhang, Yi Jiao, Xiangyi Wang, Hongjin Du, Ruichen Jiang, Di Liu, Yajing Xu, Qian Guan, Mengqian Lu

**Affiliations:** ^1^School of Acupuncture-Moxibustion and Tuina, Beijing University of Chinese Medicine, Beijing, China; ^2^Tuina and Pain Management Department, Dongzhimen Hospital of Beijing University of Chinese Medicine, Beijing, China; ^3^Acupuncture Department, Oriental Hospital of Beijing University of Chinese Medicine, Beijing, China

**Keywords:** tuina, rubbing manipulation, UC, gut microbiota, proteomics

## Abstract

Tuina can effectively alleviate ulcerative colitis-related symptoms, but the mechanism of action is unknown. The purpose of this research is to explore potential pathways for the treatment of tuina through gut microbiota and proteomics techniques. Thirty-two male BALB/c mice were divided into four groups, the control, model, mesalazine, and tuina groups. The ulcerative colitis model was established by freely drinking a 3% dextran sulphate sodium solution for 7 days. The mesalazine group and the tuina group, respectively, received 7 days of mesalazine and tuina treatment. Subsequently, their body weights, feces properties, colon length, histomorphological changes, gut microbiota, and colon proteomics were determined. Body weights, disease activity index score, colon histological scores, and microbiota diversity were restored in the tuina group. At the phylum level, Firmicutes was increased and Bacteroidota decreased. At the family level, Lachnospiraceae increased and Prevotellaceae decreased. At the genus level, the Lachnospiraceae_NK4A136_group was increased. Proteomics detected 370 differentially expressed proteins regulated by tuina, enriched to a total of 304 pathways, including biotin metabolism, Notch signaling pathway, linoleic acid metabolism, and autophagy. Tuina can effectively improve the symptoms of weight loss, fecal properties, and colon inflammation in ulcerative colitis mice and restore the gut microbiota diversity, adjusting the relative abundance of microbiota. The therapeutic effects of tuina may be achieved by modulating the signaling pathways of biotin metabolism, Notch signaling pathway, linoleic acid metabolism, and autophagy.

## Introduction

Ulcerative colitis (UC) is a chronic inflammatory disease of the colon and small intestine caused by a combination of genetic background and environmental factors (De Souza and Fiocchi, 2016). The disease is characterized by abdominal pain, diarrhea, rectal bleeding, internal cramps of the pelvis, and weight loss. The prevalence of UC exceeds 0.3%, and the incidence of pediatric UC is steadily increasing (Ng et al., 2017; Sýkora et al., 2018; Windsor and Kaplan, 2019). The development of UC is highly associated with genetic and environmental factors. These causes together with changes in the gut microbiota drive the chronic immune-mediated inflammatory response (Ni et al., 2017). The terminal ileum and colon are the most severe inflammatory sites of UC, as well as the sites with the highest concentration of gut microbes. There is an epithelial cell layer that separates the intestinal immune system and the microbiota. When the gut microbiota is imbalanced, it can easily break the intestinal barrier and affect the physiological function of the immune system (Conrad et al., 2017). Although we do not know whether the gut microbiota is a cause or a consequence of the pathogenesis of UC, changes in the microbiota are certainly consistent with the condition or prognosis of the disease, which is supported by both clinical trials and animal studies (Mukhopadhya et al., 2012).

Complementary and alternative therapies, such as tuina, acupuncture, and moxibustion, are widely used in the treatment of UC. Tuina is a therapy that treats diseases by applying pressure with direction, depth, and intensity to the skin, muscles, and joints of the body through a variety of manipulations. It has been widely used in the treatment of a variety of diseases, such as the musculoskeletal system, nervous system, etc.(Fan et al., 2021; Huang et al., 2022). In recent years, it has shown a good clinical effect on digestive system diseases and can improve symptoms such as constipation, diarrhea, pain, and dyspepsia caused by intestinal diseases (Bu et al., 2020; Fang et al., 2021). A clinical study showed that tuina can effectively improve the visual analog score and reduce pain caused by UC compared with mesalazine treatment (Yu et al., 2019). Studies have shown that between 20 and 40% of UC patients used tuina to alleviate the unbearable symptoms (Rawsthorne et al., 2012; Oxelmark et al., 2016). However, the effective mechanism of tuina therapy is still in its infancy, several studies found that tuina can down-regulate the expression levels of tumor necrosis factor-α, interleukin (IL)-6, and IL-10 to reduce inflammation, suggesting that tuina plays an effective role in immune or inflammatory diseases (Mori et al., 2004; Crane et al., 2012; Negahban et al., 2013; Barbe et al., 2021). Furthermore, enrichment for diversity and beneficial bacterial community was observed in rats after tuina treatment, it is indicated that tuina exerts an immunomodulatory effect by modulating the microbiota (Zhu et al., 2020).

Here, to explore the potential mechanisms of action for UC treatment using tuina, we combined the gut microbiota and proteomics to investigate whether tuina can alter the dysbiosis of the gut microbiota and regulate the expression of colon proteins in UC model mice. We reported that mesalazine and tuina treatment restored the lost diversity and composition after modeling, with an increased abundance of beneficial taxa associated with the alleviation of inflammatory symptoms. We identified 247 differentially expressed proteins (DEPs) regulated by mesalazine and 370 DEPs regulated by tuina and then performed bioinformatic analyzes. This study preliminary explored the potential mechanisms of tuina for the treatment of UC and provided a basis for follow-up studies.

## Materials and methods

### Animals and ethical approval

In this experiment, 32 9-week-old male BALB/c mice weighing 20 ± 1 g each were purchased from SPF (Beijing) biotechnology Co., LTD. (Beijing, China) for this experiment, the certification number is SCXK (JING) 2019–0010. The mice received sterile maintenance feed, with free access to drinking water. These animals were housed at 25 ± 0.5°C and relative humidity of 60%–70%, and the padding was changed twice a week. All animal experimental procedures were following the principles of the local animal ethics committee (The review number is BUCM-4-2,021,112,601-4,074).

### Experimental UC model induction

**Figure 1:**
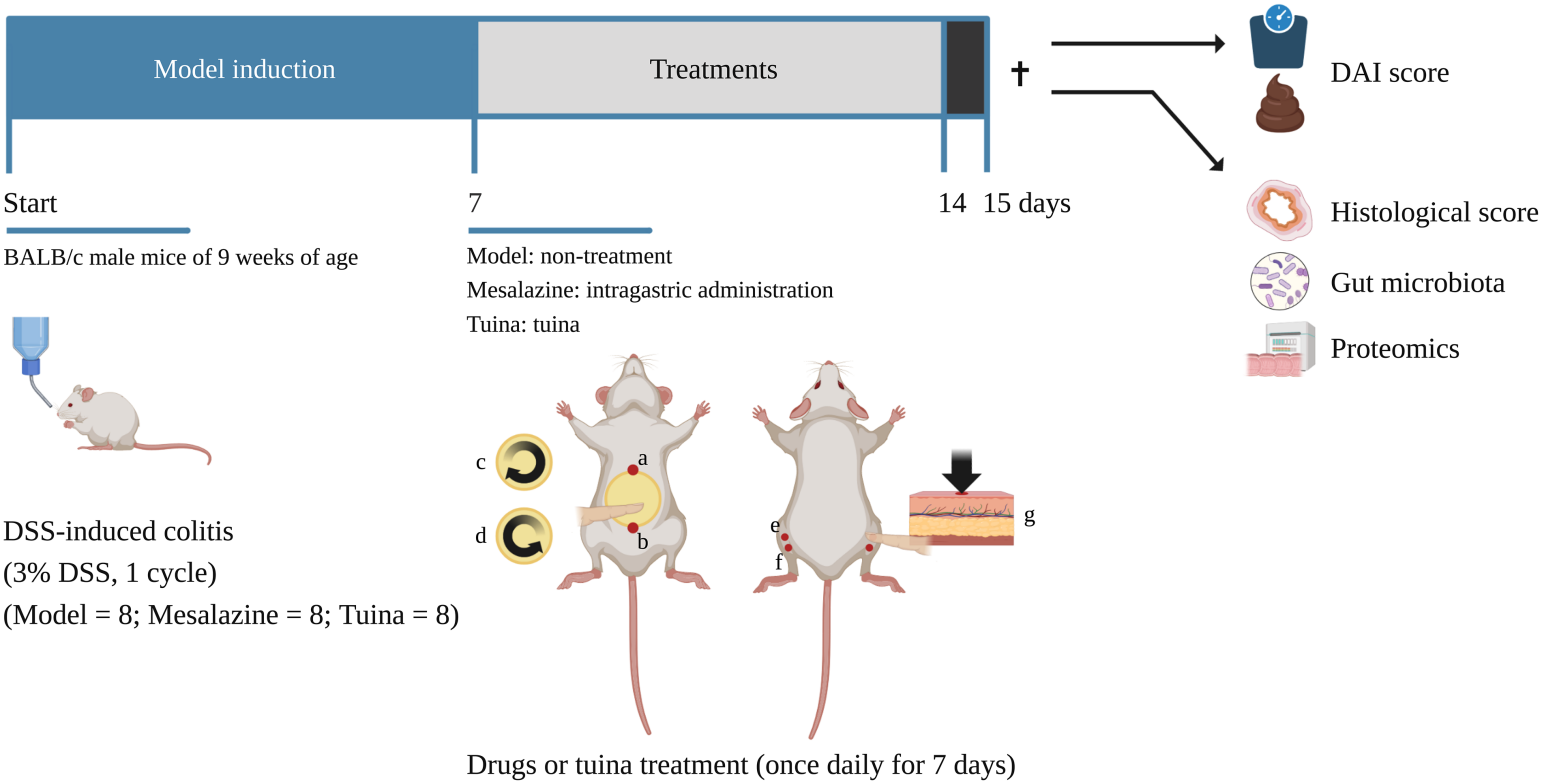
Timeline of the experiment. ✝, time point of sacrifice, the arrow above indicates before the sacrifice and the arrow below indicates after the sacrifice. a and b form the abdomen operation area. a, RN 15; b, RN 4. c and d indicate the direction of the rubbing manipulation. (c), clockwise for 3 min; (d), counterclockwise for 3 min. (e), ST 36 (bilateral); (f), ST 37 (bilateral). g indicates the direction of the pointing manipulation [images with permission created with BioRender. https://biorender.com (2022)].

After 3 days of acclimatization, at time point P1, the 32 mice were randomly assigned to the control group, the model group, the mesalazine group and the tuina group, 8 per group. Mice in the control group received distilled water. And experimental UC models were induced by freely drinking 3% dextran sulphate sodium (DSS) solution (w/v in distilled water; MPBIO, Canada) for 7 days in the other three groups (Figure 1).

### Experimental treatment

After 7 days of DSS solution induction, at time point P8, all groups received distilled water. The mesalazine group was treated with a once daily mesalazine solution (intragastrically; 500 mg/kg; IPSEN, China) solution for 7 days. The tuina group received a 10-min tuina treatment per day for 7 days (Figure 1).

### Tuina treatment

During acclimatization feeding, the manipulator performed daily handling to reduce animal stress. The tuina protocol consisted of two parts: rubbing the abdomen and pointing. The procedure was performed as follows (Figure 1): The manipulator held a mouse in the palm. First, set RN 4, and RN 15 as the operating area (the abdomen). The abdomen was exposed, and circular rubbed the abdomen with the thumb was in a clockwise direction for 3 min (100 times per minute), then operated counterclockwise for 3 min, 6 min in total, with the force of 4 N (Liu, 2006). Second, the index finger pointed bilateral ST 36 and ST 37, 1 min per acupoint, 4 min in total, with the force of 4 N (Huizhu et al., 2021). The manipulator had been trained prior to the experiment. Pressure and frequency are controlled by the fingerTPS II wireless pressure measurement system (Pressure Profile System, US).

### Evaluation of the disease activity index score and colon sample collection

At time point P15, all animals were assessed for the disease activity index (DAI) score according to the following criteria (Table 1). Then, all groups were sacrificed by IP with 2% pentobarbital. Immediately separated the colon from the rest of the abdominal tissue. Removed the entire colon between the anus to the cecum, and measured the colon length. Furthermore, 2 cm of the distal colon sample was taken for hematoxylin–eosin (HE) staining and another 2 cm for 16 S rDNA bioinformatic analysis. Each mouse collected the same segments. Meanwhile, the colon contents were rapidly removed and stored at −80°C for further analysis.

**Table 1:** Disease activity index score criteria.

Points	Body weight	Feces properties	Occult blood
0	No loss	Normal	−
1	1%–5% loss	Soft	+
2	6%–10% loss	Wet and soft	++
3	11%–15% loss	Watery and semi-loose	+++
4	16% and over loss	Watery and loose	Gross

### He staining and evaluation of the colon histological score

The colon tissues were fixed in a 4% paraformaldehyde fixing solution at 4°C for 48 h. The tissues were then embedded in paraffin and sectioned, the thickness was 4 μm. The glass slides were hydrated at 65°C for 1 h and stained with HE solution (Solarbio, China), then observed and photographed through the microscope (NIKON, Japan). Colon histological scores were graded according to the following standard of tissue injury (Table 2).

**Table 2:** Colon histological score standard.

Points	Inflammation	Intestinal mucosa
0	No	No interstitial edema, and no hurt in the mucosa
1	Slight	Interstitial edema, and mucosal hurts
2	Moderate	Interstitial edema, and mucosal and submucosal hurts
3	Severe	Interstitial edema, and transmural hurts

### 16 S rDNA bioinformatics analysis

Gut microbiota analysis was performed by the 16 S rDNA bioinformatics approach. Briefly, the genomic DNA of the microbial community in fecal samples was extracted by a soil DNA kit (Omega Bio-Tek, US). After qualifying for the concentration and purity test, proceed to the next operation. The V3-V4 hypervariable region of bacterial 16S rRNA gene was amplified by forwarding primer 338 and reverse primer 806, and the primer sequences are 5′-ACTCCTACGGGAGGCAGCAG-3′ and 5′-GGACTACHVGGGTWTCTAAT-3′. Then triple PCR 16S rRNA gene amplification was performed. According to the standard protocol, the purified amplicons were pooled and end-sequenced using the MiSeq PE 300 (Illumina, US) platform. Then, using FastQ to quality filter the raw reads of 16S rRNA gene sequencing, and using Fast Length Adjustment of Short reads to merge (Magoč and Salzberg, 2011; Chen et al., 2018). The UPARSE was used to cluster the operational taxonomic units (OTUs) with 97% similarity, and the chimeric sequences were identified and deleted (Edgar, 2013). Using 0.7 as the confidence threshold, the 16S rRNA database was then analyzed by the RDP Classifier for each representative OTU sequence (Wang et al., 2007).

### Data independent acquisition quantitative proteomics analysis

The DIA quantitative analysis process includes 5 steps: total protein extraction, protein digestion, high pH RP-UPLC separation, liquid chromatography-mass spectrometry analysis, and protein identification (Law and Lim, 2013). Briefly, the colon tissue samples were incubated on ice in protein lysis buffer (with 8 M urea, 1% SDS, and protease inhibitor) for 30 min, then, all samples were centrifuged for 30 min at 4°C, set the speed to 16,000 g, and determined the protein supernatant concentration using the bicinchoninic acid method. The protein digestion process includes 3 steps: resuspension was performed with triethylammonium bicarbonate buffer (100 mM, Sigma-Aldrich, and Germany). Then the reduction was performed with Tris (2-carboxyethyl) phosphine buffer (10 mM, Sigma-Aldrich, and Germany) for 60 min at 37°C. At last, alkylation was performed with iodoacetamide buffer (40 mM, Sigma-Aldrich, and Germany) for 40 min in darkness at room temperature. After the samples were centrifuged for 20 min at 4°C and set the speed to 10,000 g, the collected pellet was resuspension and incubation at 37°C overnight after adding trypsin. The trypsin-digested peptides were vacuum-dried and resuspended in UPLC loading buffer, then fractionated into fractions to increase proteome depth. Online analysis of the redissolved peptides using a nanoflow liquid chromatography–tandem mass spectrometry method with the EASY-nLC system (Thermo Fisher Scientific, USA). Ran the Q Exactive HF-X in DIA mode with variable isolation windows, set with 40 windows, each overlapping by 1 m/z. Last, use the default settings of Spectronaut (Version 14; Biognosys AG) for analysis of DIA data files. A cutoff value of 1% was used as the Q-value (FDR) at the precursor level and the protein level. The six highest intensity peptides were used for quantification.

### Statistical analysis

Data from this study were statistically analyzed using GraphPad Prism (Version 9.3.1). Data were presented by mean ± standard error (SEM). A two-way ANOVA and Tukey’s multiple comparisons were used to test the significance differences of the data and *p* < 0.05 was considered statistical significance. Differential abundance testing between groups and *post hoc* tests were performed using the Benjamini-Hochberg method and Tukey’s multiple comparisons. The Chao diversity index was used to measure Alpha diversity. The binary-jaccard distance and principal coordinates analysis (PCoA) was used to analyze Beta diversity. The metabolic functional prediction analysis was performed using the PICRUSt analysis platform. In this study, the identification thresholds for DEPs were selected as fold change >1.2 or < 0.83 and *p*-value <0.05. Using the GO (http://geneontology.org/) and KEGG pathway (https://www.kegg.jp/kegg/) platforms for DEPs annotation and enrichment bioinformatics analysis (Mi et al., 2019).

## Results

### Tuina improves UC-related symptoms in DSS-induced model mice

**Figure 2:**
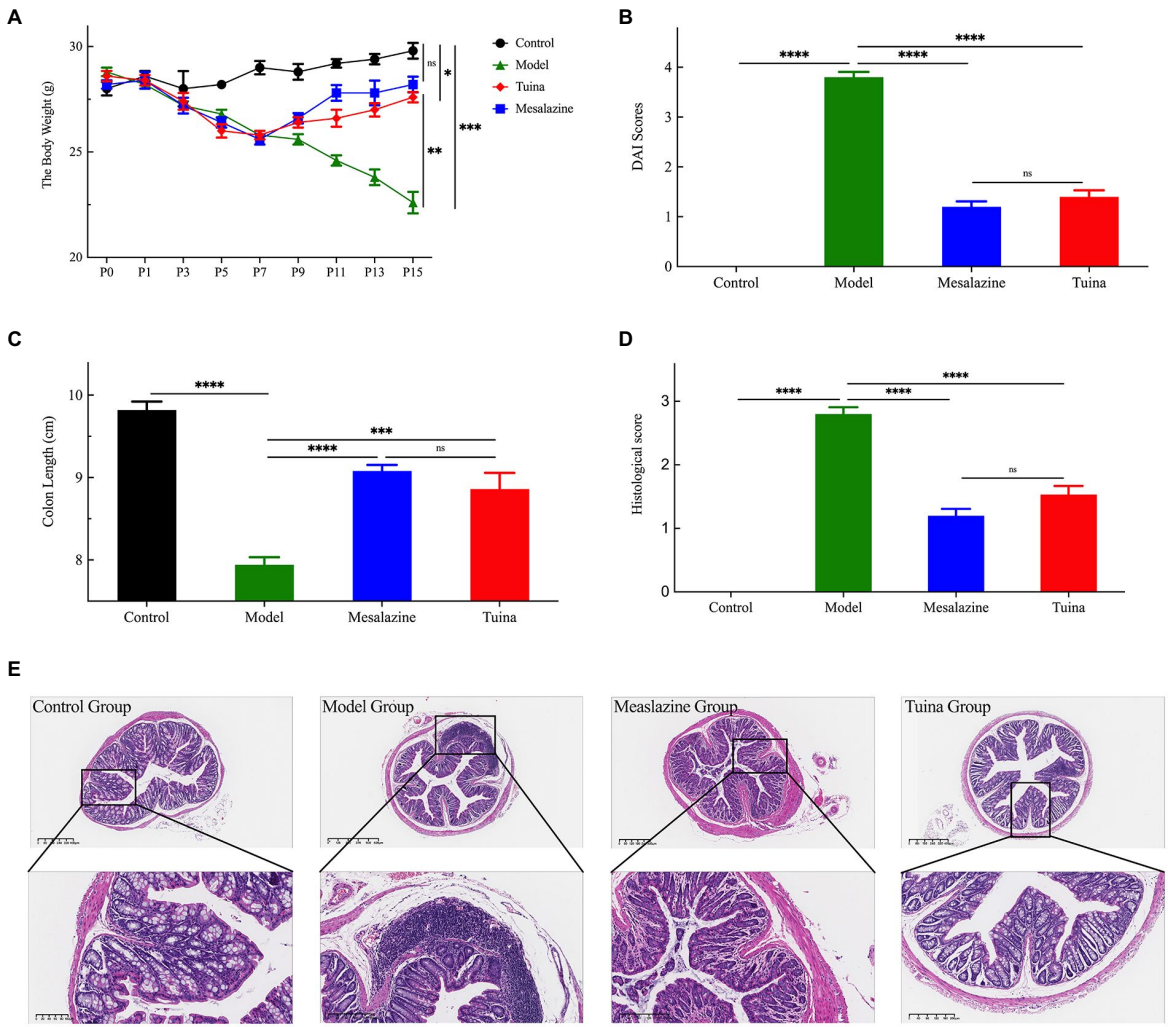
Tuina treatment prevents UC-related symptoms in model mice. **(A)** Changes in body weight of all groups throughout the study. **(B)** DAI score for each group. **(C)** The colon length of all groups. **(D)** Colon histological score of all groups. **(E)** The HE staining analysis, representative images are shown. ^a^*p* < 0.05, vs. control group; ^b^*p* < 0.05, vs. model group; ^c^*p* < 0.05, vs. model group.

The body weights of the mice were measured every 2 days during the experiment (Figure 2A). As the experiment proceeded, the control group gradually gained weight. The model, mesalazine, and tuina group gradually lost weight during model induction, and with statistical differences at P5 (^a^*p* = 0.015). At time points P8 to P15, the model group maintained the trend of weight loss. In contrast, treatments with mesalazine or tuina showed effects (^b^*p* = 0.010; ^c^*p* = 0.007). Mice that received tuina showed a slightly lower weight but no statistical difference compared with the mesalazine group (*p* = 0.208). Along with pathological weight loss, changes in the properties of stool, fecal occult blood, or even bloody stool are also caused by DSS (Figure 2B). The stool properties in the control group were consistently normal, with a DAI score of zero. However, the model group showed persistent occult blood in the feces started on the 3rd day after model induction, and bloody stools appeared on the 9th day after modeling. After all, when the treatments were completed, the stool properties of the mesalazine group and the tuina group returned to normal and without occult blood (^b^*p* < 0.000; ^c^*p* < 0.000). Although the DAI score in the mesalazine group was better than the tuina group, there was no statistical between the two groups (*p* = 0.496).

DSS-induced DAI score changes can also be reflected by changes in colon lengths (Figure 2C). The colons of the model group were the shortest among these four groups, and the control group was the longest. A comparison of colon lengths showed that the mesalazine group was longer than the tuina group, but there was no statistical difference (*p* = 0.612). However, 2 groups were statistically significant different when compared with the model group (^b^*p* < 0.000; ^c^*p* < 0.000). The degree of change in the morphological structure of the colon before and after treatments was assessed using the colon histological score (Figures 2D,E). The model group had the highest colon histological score and the most severe colon morphological abnormalities, such as colon inflammation and destruction of the epithelial barrier. HE staining showed that the model group had crypt injury, goblet cells, and epithelial cell loss, and infiltration of transmural inflammatory cells in the mucosa, submucosa, or glands. After tuina treatment, the colon histological score was significantly reduced (^b^*p* < 0.000), at the same time, reduced colon inflammation, preservation of structural integrity, and promotion of cell recovery can be observed. The results of the mesalazine group were similar to the tuina group (*p* = 0.101). Altogether, these results indicated that tuina could improve damages, reduce inflammation, and protect the structure of the colon.

### Variation in community abundance between the control group and model group

**Figure 3:**
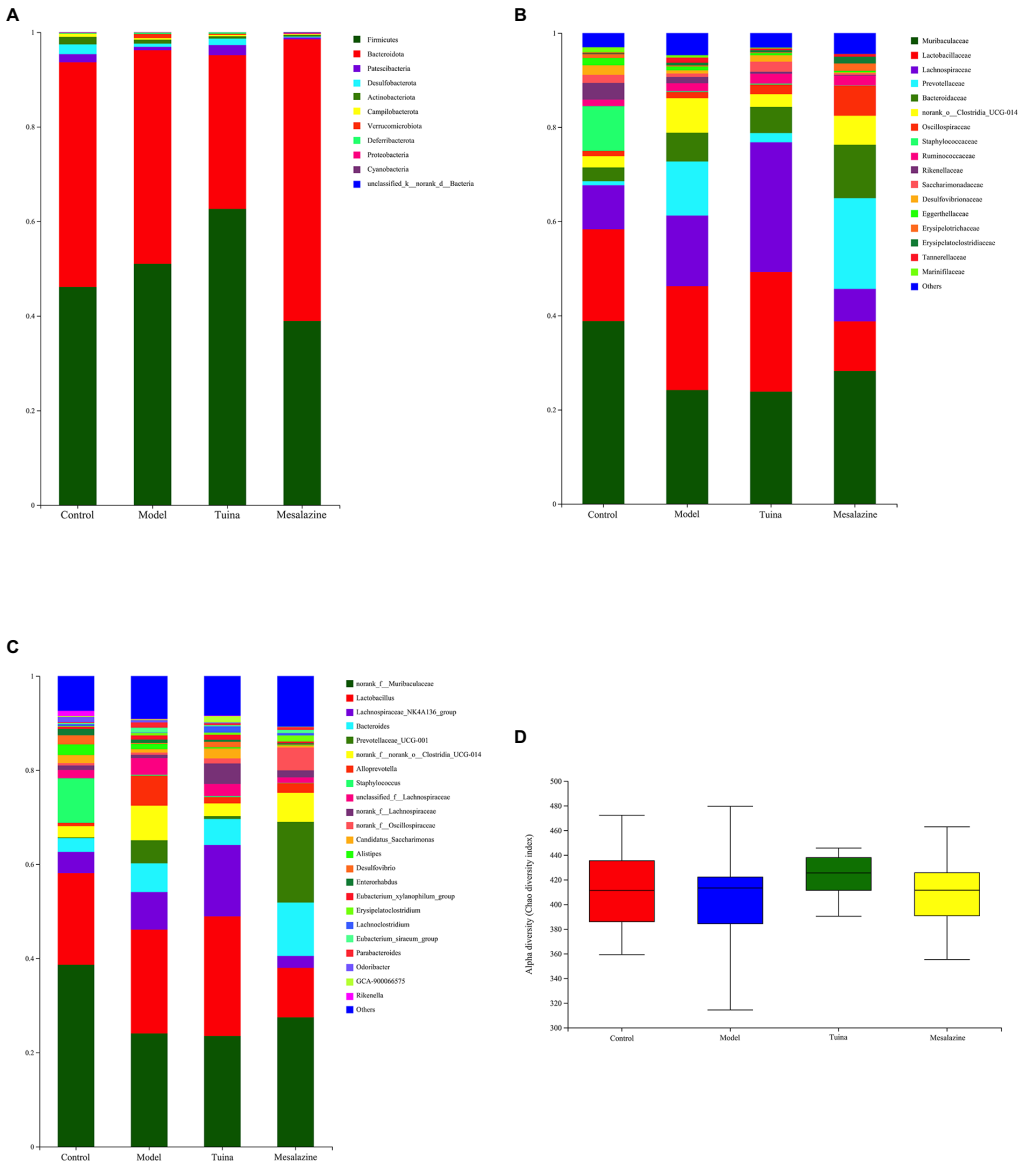
Analysis and comparison of dominant phylum, family, and genus in different groups. **(A)** At the phylum level, the horizontal coordinate represents the group and the vertical coordinate represents the percent of community abundance; **(B)** At the family level; **(C)** At the genus level; **(D)** The alpha diversity (Chao diversity index).

Compared with the control group, there was a significant difference in the gut microbiome of the model group. At the phylum level, the predominant phyla of all groups are composed of Firmicutes and Bacteroidota, and the model mice showed an increased relative abundance of Verrucomicrobiota and a decreased relative abundance of Patescibacteria, Desulfobacterota, Actinobacteriota, and Campilobacterota (Figure 3A). At the family level, the abundance of Lachnospiraceae, Prevotellaceae, and Bacteroidaceae increased and Muribaculaceae, Staphylococcaceae and Rikenellaceae decreased (Figure 3B). At the genus level, the relative abundance of Staphylococcus, Desulfovibrio, and Rikenella decreased and Bacteroides, Lachnospiraceae_NK4A136_group, Prevotellaceae UCG-001, and Alloprevotella increased (Figure 3C). Overall, the alpha diversity of the model group was reduced (Figure 3D).

### Variation in community abundance between DSS-induced model mice with tuina treatment or mesalazine treatment

Compared with the model group, at the phylum level, the relative abundance of Patescibacteria and Desulfobacterota increased and Actinobacteriota and Verrucomicrobiota decreased in the tuina group. In the mesalazine group, Proteobacteria and Cyanobacteria increased, Desulfobacterota, Actinobacteriota, and Verrucomicrobiota decreased (Figure 3A). At the family level, Lachnospiraceae and Saccharimonadaceae increased, and Rikenellaceae and Prevotellaceae decreased in the tuina group. Prevotellaceae, Bacteroidaceae, and Oscillospiraceae increased and Lachnospiraceae and Rikenellaceae decreased in the mesalazine group (Figure 3B). At the genus level, Lachnospiraceae_NK4A136_group and Candidatus_Saccharimonas increased, Prevotellaceae UCG-001, Alloprevotella, and Parabacteroides decreased in the tuina group. The relative abundance of Bacteroides and Prevotellaceae UCG-001 increased, and Lactobacillus, Lachnospiraceae_NK4A136_group, Alloprevotella and Parabacteroides decreased in the mesalazine group (Figure 3C). Mesalazine and tuina treatment can help restore the alpha diversity index to the gut microbiota (Figure 3D).

### Tuina restored the microbiota biodiversity of DSS-induced model mice

**Figure 4:**
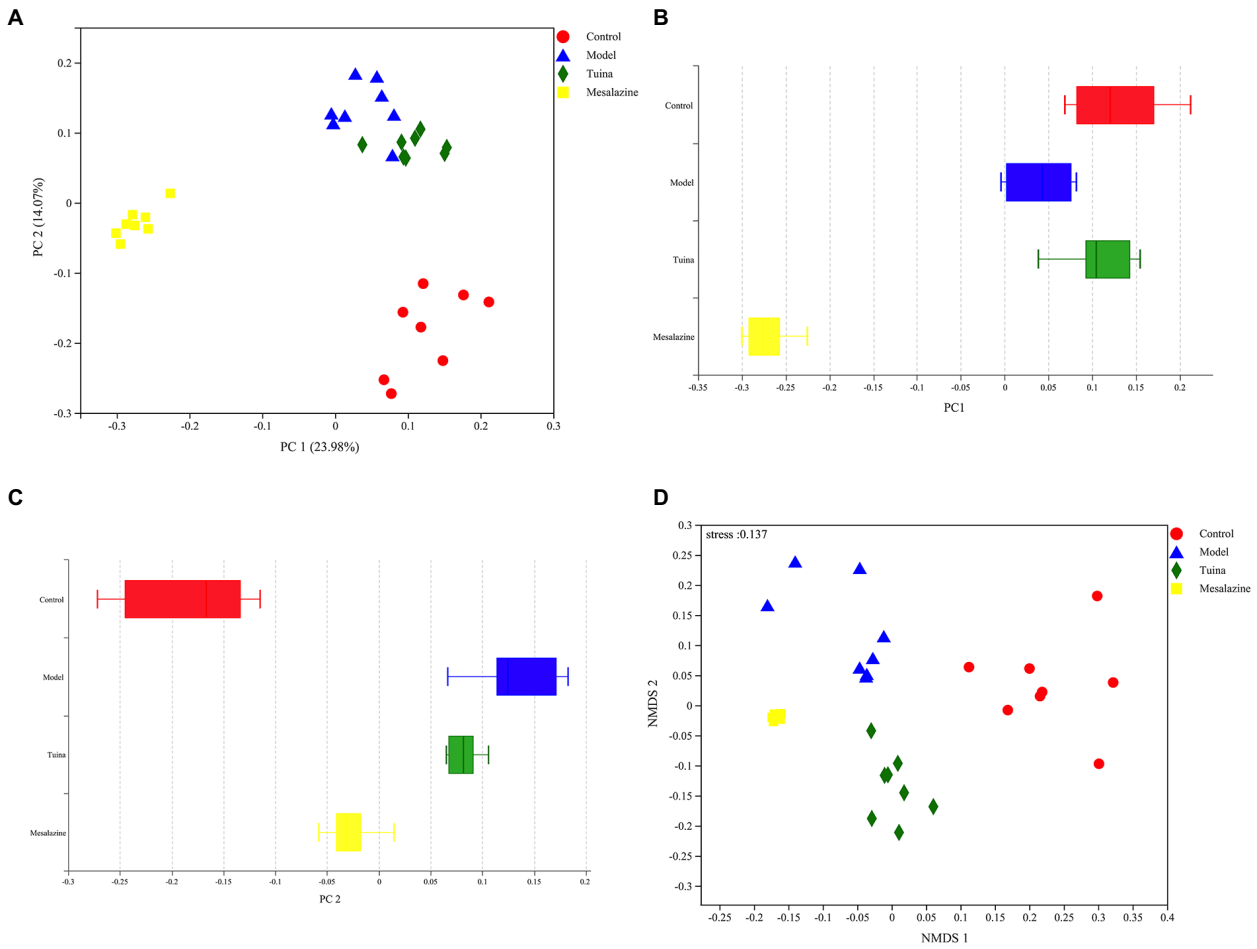
Beta diversity analysis of PCoA and NMDS. **(A)** PCoA between groups, the horizontal coordinate represents PC 1 and the vertical coordinate represents PC 2; **(B)** Distribution and dispersion of the groups on the PC 1 axis; **(C)** Distribution and dispersion of the groups on the PC 2 axis; **(D)** NMDS between groups.

The results of beta diversity showed that 4 groups were clustered separately in the PCoA, suggesting that modeling and intervention were the primary factors that affected community differences. There were very small areas of overlap between the model and tuina groups, this suggested that the tuina treatment may not be as effective as the mesalazine treatment, which is consistent with other results from this study (Figure 4A). However, on the PC 1 axis, the diversity of the tuina group was similar to that of the control group (Figure 4B). And on the PC 2 axis, the tuina group had good intragroup aggregation (Figure 4C). The merits of the results of the nonmetric multidimensional scaling (NMDS) analysis tested with the stress value, and a stress value of 0.137 represented a certain degree of explanatory significance, which indicated that the species composition was not similar between the 4 groups of samples (Figure 4D).

### The PICRUSt functional prediction analysis of microbial communities

**Figure 5:**
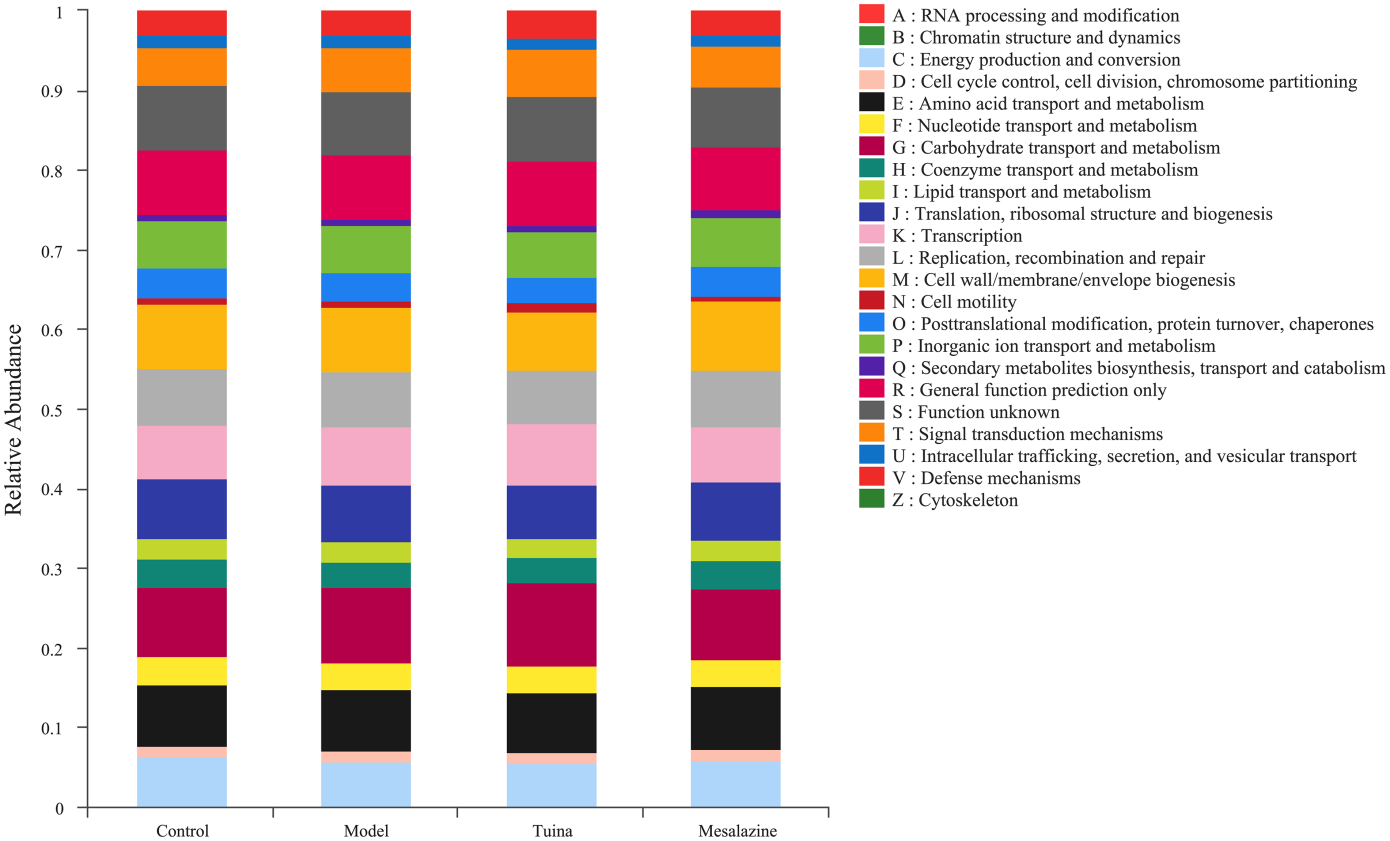
Functional abundance of COG. The vertical coordinate represents the relative abundance of the COG function of each group.

The functional abundance of metabolic COG in this community was deduced from the 16S compositional data utilizing the PICRUSt analysis platform. The metabolic COG functions of the colony were mainly focused on the transport and metabolism of carbohydrate, amino acid, and inorganic ion, and energy production and conversion (Figure 5). We suggested that the gut microbiota may play a positive role through the signaling pathways of carbohydrate, amino acid, inorganic ion, and signal transduction.

### DIA quantitative proteomics analysis of the colon samples

**Figure 6:**
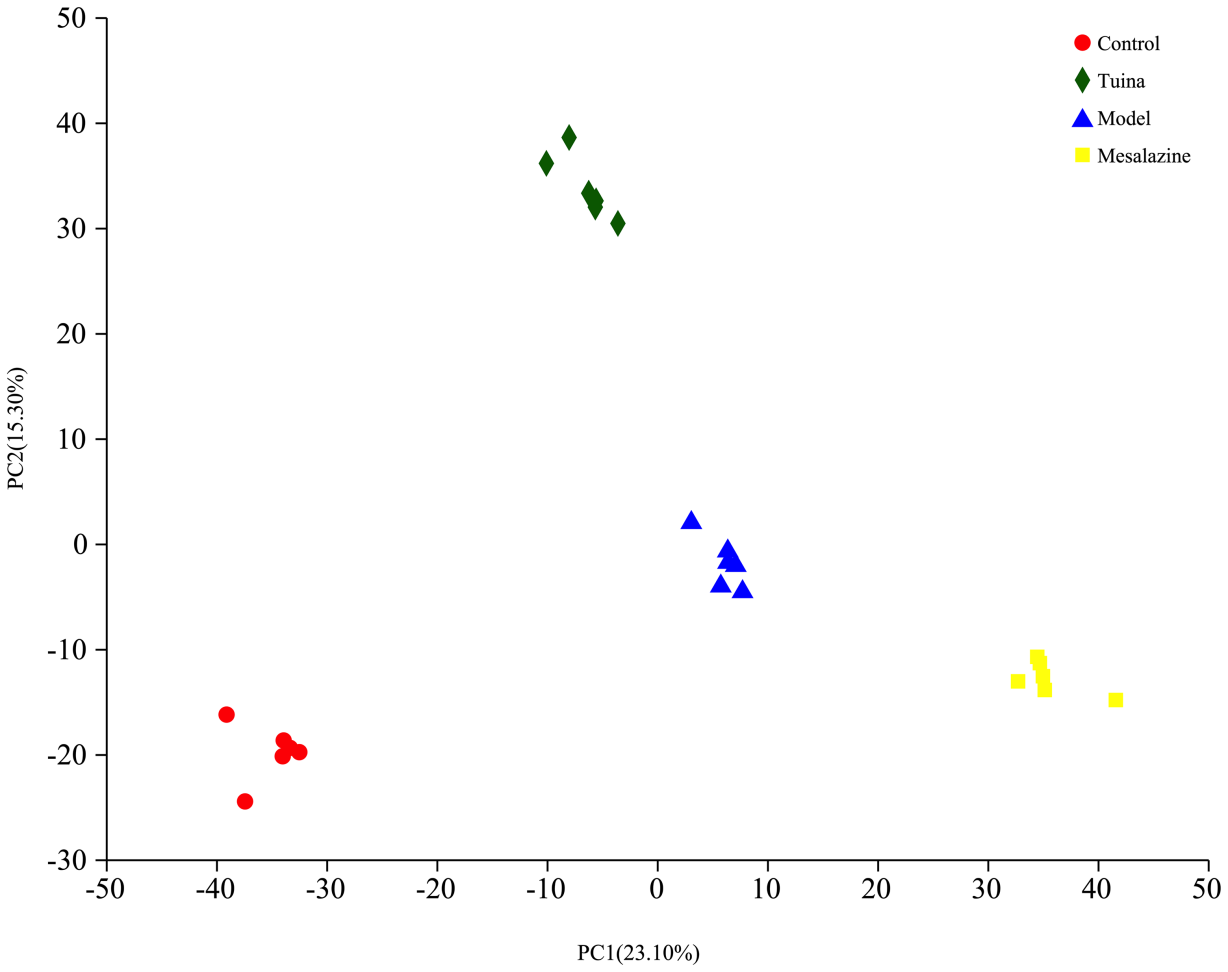
PCA analysis and volcano plots. The horizontal coordinate represents PC 1 and the vertical coordinate represents PC 2.

Quantitative proteomics analysis of DIA and bioinformatics analysis can further elucidate the potential regulatory mechanisms of tuina treatment on UC. The principal component analysis (PCA) showed excellent sample aggregation in each group, and the distance was long between the sample points in each group, indicating that the four groups were not similar to each other, the model was successfully established, and tuina or mesalazine treatment was effective (Figure 6). Proteomics analysis identified 6,294 quantifiable proteins, of which 987 DEPs in the model group vs. control group, 589 were down-regulated and 398 were up-regulated. Tuina treatment had 292 down-regulated DEPs and 578 up-regulated DEPs, for a total of 870 DEPs compared with the model group, then 843 DEPs (413 down-regulated and 430 up-regulated) were found in the mesalazine group vs. the model group.

### DEPs regulated by tuina or mesalazine treatment

**Figure 7:**
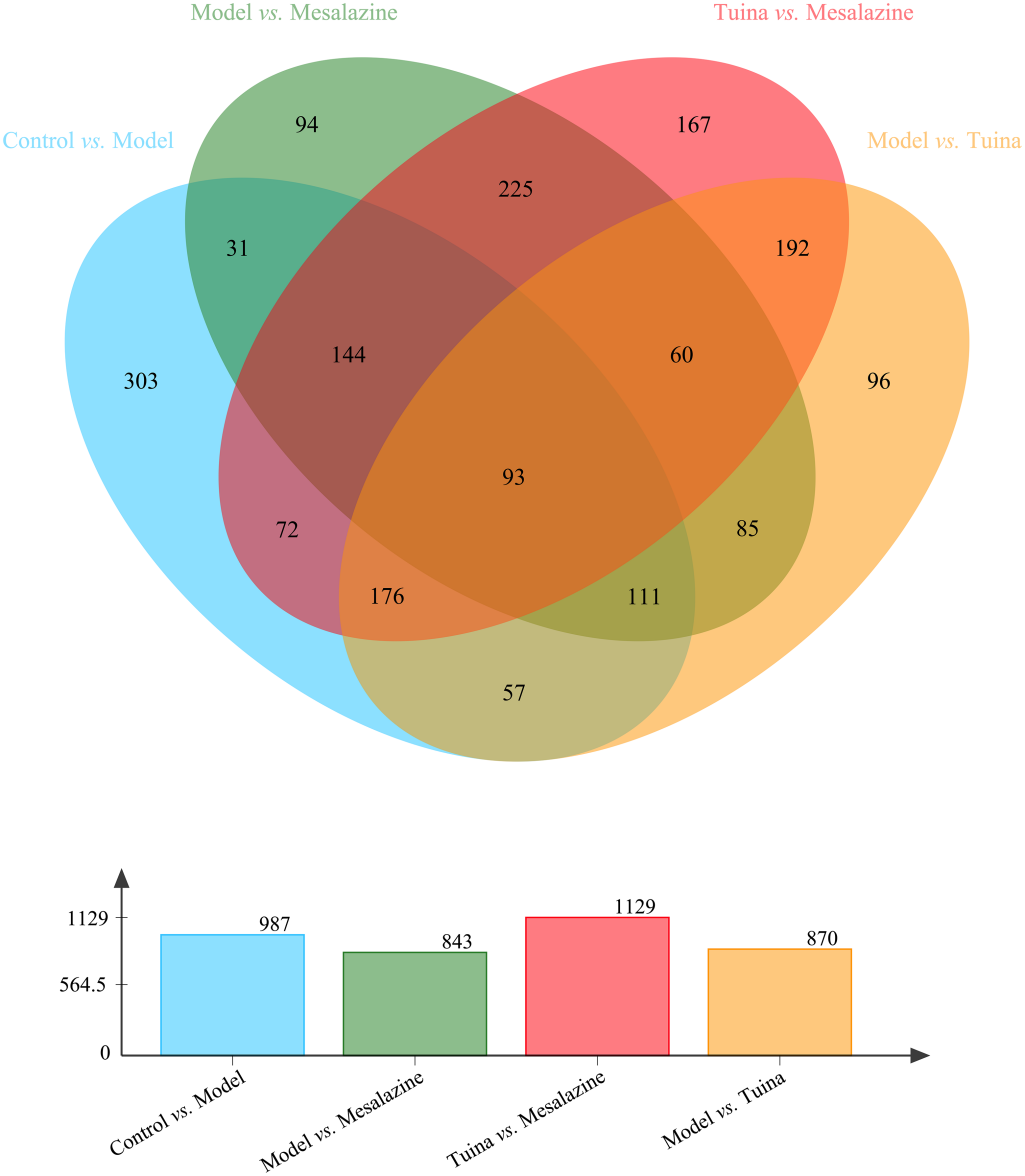
Venn analysis among each group. The bar diagram below represents the number of DEPs compared between the two groups.

To understand the unique mechanism of tuina, it is necessary to identify the DEPs that are regulated by tuina. There were 437 overlapping DEPs between the control group vs. model group and the model group vs. tuina group, of which 370 DEPs were regulated by tuina. And there were 379 overlapping DEPs between the control group vs. model group and the model group vs. mesalazine group, with 247 DEPs regulated by mesalazine treatment (Figure 7).

### Bioinformatics analyses of DEPs regulated by tuina or mesalazine

**Figure 8:**
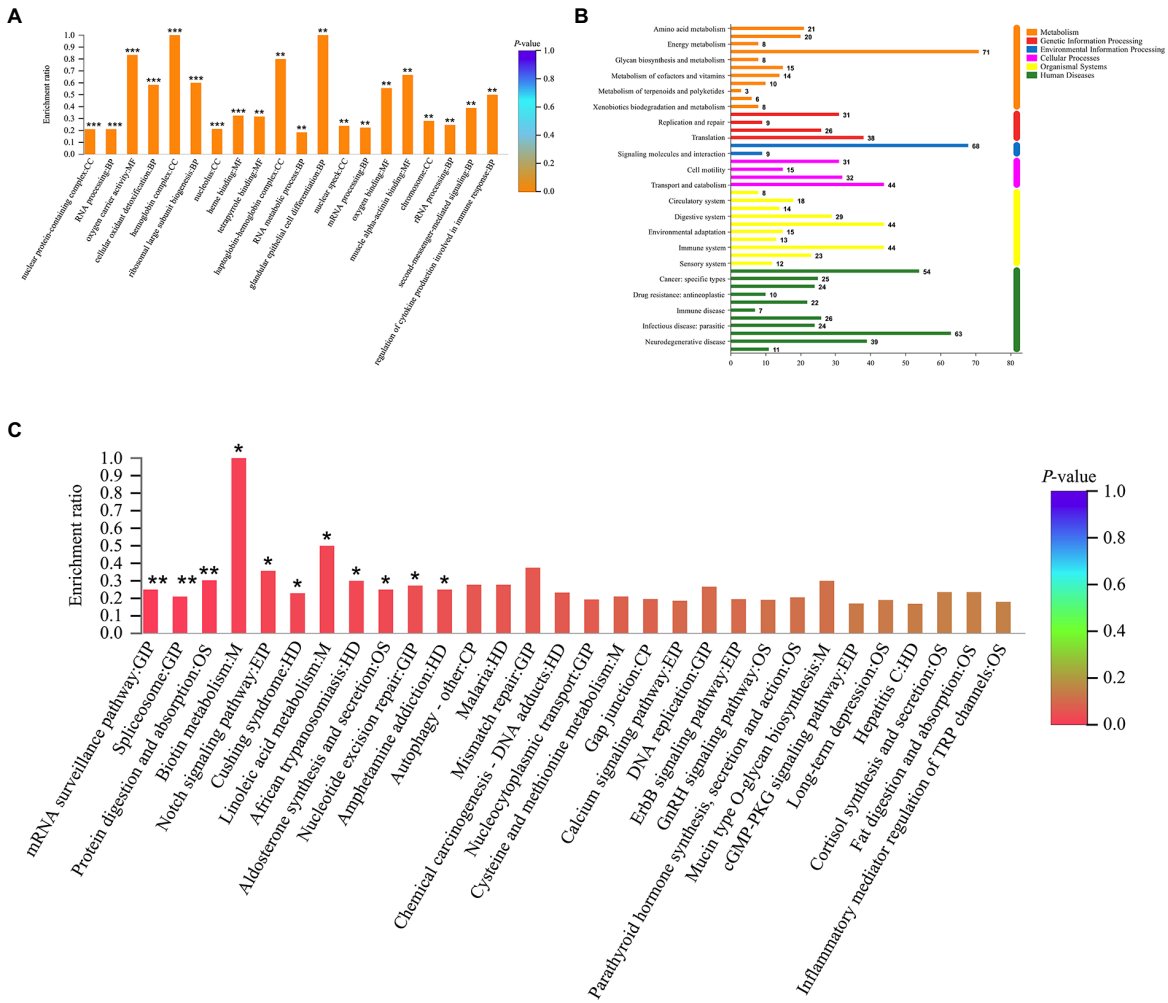
GO and KEGG analyses based on DEPs regulated by tuina treatment. **(A)** GO enrichment analysis, the abscissa represents the GO description and the Go term (level 1) and the ordinate represents the enrichment ratio, different colors represent statistical differences of enrichment; **(B)** KEGG annotation histogram, the horizontal coordinate represents the number of proteins, and the vertical coordinate represents KEGG pathways, different colors represent pathway categories; **(C)** KEGG enrichment analysis, the horizontal coordinate represents the pathway name and the vertical coordinate represents the enrichment ratio, different colors represent statistical differences of enrichment.

**Figure 9:**
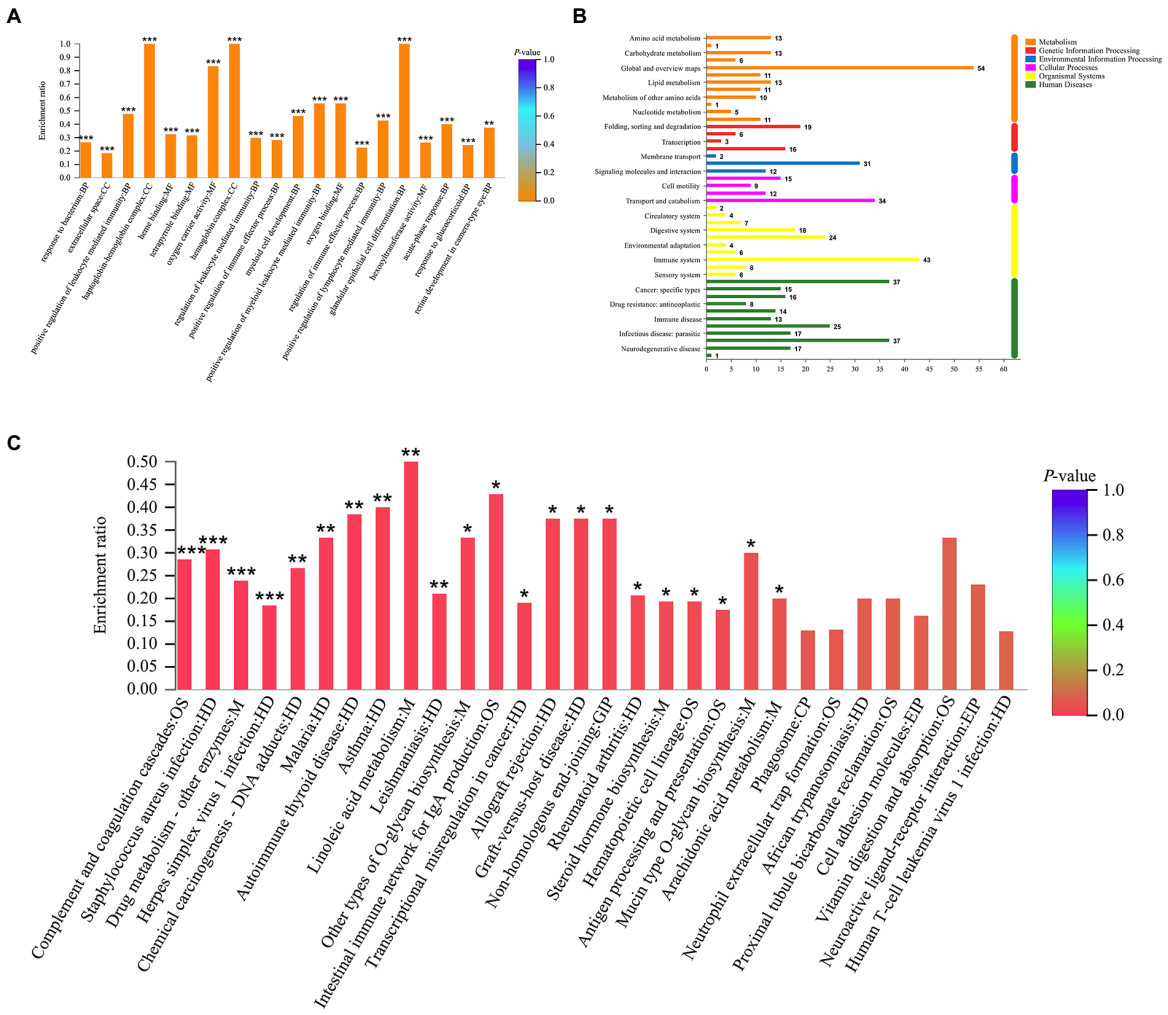
GO and KEGG analyses based on the DEPs regulated by mesalazine treatment. **(A)** GO enrichment analysis; **(B)** KEGG annotation histogram; **(C)** KEGG enrichment analysis.

GO enrichment analysis revealed a total of 265 GO terms based on 370 DEPs regulated by tuina treatment, including glandular epithelial cell differentiation, oxygen carrier activity, regulation of cytokine production involved in immune response, etc. (Figure 8A). There were 323 GO terms based on 247 DEPs regulated by mesalazine treatment, including positive regulation of leukocyte mediated immunity, response to bacterium, and positive regulation of lymphocyte leukocyte mediated immunity (Figure 9A). KEGG annotation analysis showed that there were 304 pathways based on tuina-regulated DEPs, the level 1 pathway categories with the highest number of enriched proteins were human diseases (305 proteins), organismal systems (220 proteins), and metabolism (184 proteins), level 2 pathways including signal transduction, infectious disease: viral, transport and catabolism, endocrine system, and immune system (Figure 8B). KEGG enrichment analysis showed level 3 pathways, and tuina-regulated pathways included biotin metabolism, Notch signaling pathway, linoleic acid metabolism, and autophagy (Figure 8C). There were 290 pathways based on mesalazine-regulated DEPs, and the level 1 pathway categories with the highest enriched proteins were human diseases (199 proteins), metabolism (149 proteins), and organismal systems (122 proteins), level 2 pathways including the immune system, infectious disease: viral, transport and catabolism, and infectious disease: bacterial (Figure 9B). Level 3 pathways included the intestinal immune network for IgA production, vitamin digestion and absorption, arachidonic acid metabolism, and mucin type O-glycan biosynthesis (Figure 9C).

## Discussion

Due to the unclear pathogenesis of UC, modern medicine cannot find effective therapeutic targets and methods. Tuina is gradually applied to the complementary alternative treatment of UC. Combined with the gut microbiota and proteomics, the mechanism of tuina treatment can be elucidated. Here, we histologically assessed the extent of damage to the colon morphological structures of the colon tissue and generally explored the overall changes in body weight, stool property, blood feces, and colon length under tuina treatment, mesalazine treatment, and non-treatment conditions in the UC model. We also analyzed the structural changes and diversity of the gut microbiota by 16 s rRNA high-throughput sequencing. Finally, quantitative DIA proteomics analysis was performed on colon samples to study changes in protein expression between all groups.

The DSS-induced UC model chosen for this experiment is an acute modeling method commonly used in animal studies, which is similar to the pathology of human UC. After 7 days of drinking the DSS solution, the animals showed weight loss and gross bloody feces, and the colon tissues also showed varying degrees of ulceration, edema, transmural inflammatory cell infiltration, and epithelial cell damage under microscopic observation, which proved the success of the established model (Huang et al., 2017). The gut microbiota diversity of the model mice was reduced compared with the control group, consistent with the literature (Galipeau et al., 2021).

At present, the symptoms of UC are mainly controlled by aminosalicylic acid preparations (ASAP) and glucocorticoids. The representative drug for ASAP is mesalazine, which reduces PEG2 synthesis in the mucous membrane of the colon and inhibits neutrophil function (Nitta et al., 2002). The main types of administration include oral and rectal administration, bioavailability is approximately 30% regardless of the route of administration, and the half-life is approximately 10 h (Schwab and Klotz, 2001). However, the therapeutic effect of mesalazine depends on the maintenance of effective doses and has adverse effects on liver function. So we chose mesalazine as a drug control and the experimental results proved its effectiveness. The results showed that after the drug intervention, the fecal properties were restored and their body weight was increased compared with the model mice, and the colon tissue structure was well regained under the microscope. Bacterial diversity was also restored compared with the model group.

Tuina treatment used abdominal rubbing manipulation and pressing manipulation of ST 36 and ST 37. Abdomen rubbing manipulation is the main method, it can reduce the volume of gastric residue, reduce distension, increase bowel movements, and improve gastrointestinal functions without side effects (Dehghan et al., 2020). ST36 and ST37 belong to the stomach meridian (ST), they can reduce local inflammation, visceral hypersensitivity, and improve gastrointestinal transport (Wang et al., 2015; Qu et al., 2020). Although many clinical studies have confirmed the therapeutic effect of tuina treatment in diseases such as constipation, there is no experimental evidence of the mechanism underlying its efficacy. In recent years, research on the treatment of UC in Chinese medicine has been increasing and certain achievements have been made. For example, a single herb Patrinia villosa or a Ganluyin herbal formula can exert anti-inflammatory effects through pathways such as NF-κB (Wang et al., 2022; Xiong et al., 2022). Acupuncture has also been shown to alleviate UC-related symptoms by restoring the diversity of the gut microbiota (Wei et al., 2019). Tuina is a non-invasive intervention with minimal adverse effects. The results of the present study showed that body weight loss, intestinal inflammation, and loose and watery feces can be reversed by tuina. A detailed study of the DAI score results indicated that the mice in the tuina and mesalazine groups showed similar recovery trends. The same results were shown for morphological evidence of colon length changes and histological structure changes under microscopy. All evidence confirmed that there were no statistical differences between the tuina and mesalazine groups, but the mesalazine group was superior to the tuina group.

The results of the gut microbiology examination did not show overlap in the expression of the tuina group and the mesalazine group, and we speculated that the treatment of tuina and mesalazine achieves therapeutic effects through different mechanisms. At the phylum level, we found that tuina increased the relative abundance of Firmicutes and decreased Bacteroidota. Firmicutes account for about 30% of the total number of gut microorganisms, which together with Bacteroidota cover more than 90% of gut microbes. It can regulate energy absorption and metabolic conversion by the body and are a relatively stable species in the gastrointestinal flora (Tremaroli and Bäckhed, 2012). Firmicutes increased and Bacteroidota decreased are considered to be associated with weight gain, reduced obesity-induced low-grade inflammation, and inflammatory phenotype (Chakraborti, 2015). At the family level, we observed that tuina treatment could increase the relative abundance of the protective strain Lachnospiraceae in inflammatory bowel disease, its family members can prevent colon cancer by producing butyric acid (Ai et al., 2019; Zeng et al., 2020). These results suggest that tuina can improve nutritional intake, increase body weight and reduce intestinal inflammation in DSS-induced UC mice, reduce the expression abundance of opportunistic pathogens, for example, Prevotellaceae, and prevent cancer development, consistent with the literature on other Chinese medical treatments (Qi et al., 2018). At the genus level, it has been suggested that the Lachnospiraceae_NK4A136_group belongs to the intestinal beneficial bacteria, and the higher abundance can reduce intestinal inflammation, diarrhea, and other symptoms (Wu et al., 2021). However, it has also been suggested in the literature that the Lachnospiraceae_NK4A136_group is associated with intestinal dysfunction and a lower abundance is beneficial to maintain intestinal flora balance (Wang et al., 2021). In this study, an increased abundance of Lachnospiraceae_NK4A136_group was observed after tuina treatment and with decreased abundance in the mesalazine group.

Tuina can regulate biotin metabolism, Notch signaling pathway, linoleic acid metabolism, autophagy, etc. Biotin, a water-soluble vitamin, is involved in the immune and inflammatory response, cellular stress response (Kuroishi, 2015; Elahi et al., 2018). Patients with inflammatory bowel disease (IBD) are accompanied by a biotin deficiency (Erbach et al., 2022). Biotin deficiency is associated with severe colitis, which can be alleviated by biotin supplementation. Skupsky found that biotin supplementation could significantly improve DAI, colon length, and mucosal morphology of DSS mice, suggesting that biotin may have the potential to treat IBD (Skupsky et al., 2020). The Notch signaling pathway participates in regulating the development of intestinal epithelial cells and maintaining the stability of the internal environment (Wu et al., 2021). It was found that Notch signaling pathway genes were overexpressed in the proliferative recess of intestinal cells of DSS colitis mice (Noah and Shroyer, 2013). Inhibited differentiation of the intestinal epithelium into goblet cells and weakened intestinal mucous barrier function were associated with abnormal expression of the Notch signaling pathway (Pope et al., 2014; Lin et al., 2019). In this study, the goblet cells in intestinal epithelial cells of DSS model mice decreased, while increasing after tuina, suggesting that tuina may protect the mucus barrier by inhibiting the Notch signaling pathway. Excessive intake of linoleic acid is a risk factor for the development of IBD (Owczarek et al., 2016). The level of linoleic acid in patients with UC and CD was higher than in healthy controls (Ueda et al., 2008). Tefas et al. found that IBD patients showed significant changes in 6 lipids and 7 metabolites compared to healthy ones, and most of them belonged to linoleic acid metabolism and glycerophospholipid (Tefas et al., 2020). Autophagy is a key factor in maintaining the stability of intestinal homeostasis and can regulate the intestinal microbiota and immunity response (Larabi et al., 2020). It has been proved that the variation of autophagy-related genes can lead to apoptosis. Autophagy-related gene defects in colonic epithelial cells can affect the microbiota composition, such as Lachnospiraceae, Proteobacteria, and Cyanobacteria. This is consistent with the results we obtained for the gut microbiota (Tsuboi et al., 2015; Yang et al., 2018; Lavoie et al., 2019). The limitation of this study is that there was no molecular biological validation of the relevant inflammatory indicators and possible pathways. In the next step we want to screen among the potential pathways, design new experimental protocols, explore regulatory pathways, and clarify key protein target relationships to explain the action mechanism of tuina.

## Conclusion

In conclusion, tuina can effectively improve the DAI score in DSS-induced UC mice, relieve colitis and restore colon morphology, as well as restore gut microbiota diversity and adjust microbiota structure. The therapeutic effects of tuina may be related to modulating biotin metabolism, Notch signaling pathway, linoleic acid metabolism, and autophagy signaling pathways.

## Data availability statement

The original contributions presented in this study are publicly available. The raw data can be found on NCBI at the following link: http://www.ncbi.nlm.nih.gov/bioproject/905890 with accession number: PRJNA905890. Further inquiries can be directed to the corresponding author.

## Ethics statement

The animal study was reviewed and approved by the Animal Ethics Committee of the Beijing University of Chinese Medicine.

## Author contributions

ML and TY: study conception and design of the work. HW, ZL, XW, HD, RJ, DL, YX, and QG: data acquisition. HW and ZL: analysis and data interpretation, drafting of the manuscript. HW, ZL, YZ, and YJ: approval of the final version of the manuscript. All authors contributed to the article and approved the submitted version.

## Funding

The authors have received funding for research, writing, and publication of this paper from the National Natural Science Foundation of China (No. 81704193).

## Conflict of interest

The authors declare that the research was conducted in the absence of any commercial or financial relationships that could be construed as a potential conflict of interest.

## Publisher’s note

All claims expressed in this article are solely those of the authors and do not necessarily represent those of their affiliated organizations, or those of the publisher, the editors and the reviewers. Any product that may be evaluated in this article, or claim that may be made by its manufacturer, is not guaranteed or endorsed by the publisher.

## References

[ref1] Ai D. Pan H. Li X. Gao Y. Liu G. Xia L. C. (2019). Identifying gut microbiota associated with colorectal cancer using a zero-inflated lognormal model. Front. Microbiol. 10:826. doi: 10.3389/fmicb.2019.00826, PMID: 31068913 PMC6491826

[ref2] Barbe M. F. Panibatla S. T. Harris M. Y. Amin M. Dorotan J. T. Cruz G. E.. (2021). Manual therapy with rest as a treatment for established inflammation and fibrosis in a rat model of repetitive strain injury. Front. Physiol. 12:1925. doi: 10.3389/fphys.2021.755923, PMID: 34803739 PMC8600143

[ref3] Bu F. L. Han M. Lu C. L. Liu X. H. Wang W. G. Lai J. L.. (2020). A systematic review of Tuina for irritable bowel syndrome: recommendations for future trials. Complement. Ther. Med. 52:102504. Epub 2020/09/22. doi: 10.1016/j.ctim.2020.102504, PMID: 32951752

[ref4] Chakraborti C. K. (2015). New-found link between microbiota and obesity. World J. Gastrointest. Pathophysiol. 6, 110–119. doi: 10.4291/wjgp.v6.i4.110, PMID: 26600968 PMC4644874

[ref5] Chen S. Zhou Y. Chen Y. Gu J. (2018). Fastp: an ultra-fast all-in-one Fastq preprocessor. Bioinformatics 34, i884–i890. Epub 2018/11/14. doi: 10.1093/bioinformatics/bty560, PMID: 30423086 PMC6129281

[ref6] Conrad M. A. Wu G. D. Kelsen J. R. (2017). The Gut Microbiota and Inflammatory Bowel Disease. Pediatric Inflammatory Bowel Disease: 3rd. Cham, Switzerland: Springer International Publishing. p. 45–54.

[ref7] Crane J. D. Ogborn D. I. Cupido C. Melov S. Hubbard A. Bourgeois J. M.. (2012). Massage therapy attenuates inflammatory signaling after exercise-induced muscle damage. Sci. Transl. Med. 4:119ra13. doi: 10.1126/scitranslmed.3002882, PMID: 22301554

[ref8] De Souza H. S. P. Fiocchi C. (2016). Immunopathogenesis of Ibd: current state of the art. Nat. Rev. Gastroenterol. Hepatol. 13, 13–27. doi: 10.1038/nrgastro.2015.186, PMID: 26627550

[ref9] Dehghan M. Malakoutikhah A. Ghaedi Heidari F. Zakeri M. A. (2020). The effect of abdominal massage on gastrointestinal functions: a systematic review. Complement. Ther. Med. 54:102553. doi: 10.1016/j.ctim.2020.102553, PMID: 33183670

[ref10] Edgar R. C. (2013). Uparse: highly accurate Otu sequences from microbial amplicon reads. Nat. Methods 10, 996–998. Epub 2013/08/21. doi: 10.1038/nmeth.2604, PMID: 23955772

[ref11] Elahi A. Sabui S. Narasappa N. N. Agrawal S. Lambrecht N. W. Agrawal A.. (2018). Biotin deficiency induces Th1- and Th17-mediated Proinflammatory responses in human Cd4 + T lymphocytes via activation of the Mtor signaling pathway. J. Immunol. 200, 2563–2570. doi: 10.4049/jimmunol.1701200, PMID: 29531163 PMC5893381

[ref12] Erbach J. Bonn F. Diesner M. Arnold A. Stein J. Schröder O.. (2022). Relevance of biotin deficiency in patients with inflammatory bowel disease and utility of serum 3 Hydroxyisovaleryl Carnitine as a practical everyday marker. J. Clin. Med. 11:1118. doi: 10.3390/jcm11041118 35207391 PMC8877558

[ref13] Fan Z. Di A. Huang F. Zhao S. Qiu M. Wu C.. (2021). The effectiveness and safety of Tuina for tension-type headache: a systematic review and meta-analysis. Complement. Ther. Clin. Pract. 43:101293. Epub 2021/03/19. doi: 10.1016/j.ctcp.2020.101293, PMID: 33735636

[ref14] Fang Y.-P. Huang Y.-T. Chen D. Kan Y. Wang J.-W. Kang X.-L.. (2021). Systematic review and meta analysis on the effectiveness and safety of Tuina in treatment of functional constipation. Zhongguo Zhen Jiu 41, 691–698. doi: 10.13703/J.0255-2930.20200411-0004, PMID: 34085491

[ref15] Galipeau H. J. Caminero A. Turpin W. Bermudez-Brito M. Santiago A. Libertucci J.. (2021). Novel fecal biomarkers that precede clinical diagnosis of ulcerative colitis. Gastroenterology 160, 1532–1545. doi: 10.1053/j.gastro.2020.12.004, PMID: 33310084

[ref16] Huang Y. Ma Z. Cui Y. H. Dong H. S. Zhao J. M. Dou C. Z.. (2017). Effects of herb-partitioned Moxibustion on the Mirna expression profiles in colon from rats with Dss-induced ulcerative colitis. Evid. Based Complement. Alternat. Med. 2017:1767301. doi: 10.1155/2017/1767301, PMID: 28246536 PMC5299174

[ref17] Huang F. Xiao Z. Zhan X. Zeng P. Zhao S. Guo R.. (2022). Tuina combined with adjuvant therapy for lumbar disc herniation: a network meta-analysis. Complement. Ther. Clin. Pract. 49:101627. Epub 2022/07/20. doi: 10.1016/j.ctcp.2022.101627, PMID: 35849972

[ref18] Huizhu N. Biwei C. Qiaobin C. Shaozong C. (2021). Rules of acupoints selection in treating UC based on data mining. Acta Chin. Med. Pharmacol. 49, 45–49. doi: 10.19664/j.cnki.1002-2392.210235

[ref19] Kuroishi T. (2015). Regulation of immunological and inflammatory functions by biotin. Can. J. Physiol. Pharmacol. 93, 1091–1096. doi: 10.1139/cjpp-2014-0460, PMID: 26168302

[ref20] Larabi A. Barnich N. Nguyen H. T. T. (2020). New insights into the interplay between autophagy, gut microbiota and inflammatory responses in Ibd. Autophagy 16, 38–51. doi: 10.1080/15548627.2019.1635384, PMID: 31286804 PMC6984609

[ref21] Lavoie S. Conway K. L. Lassen K. G. Jijon H. B. Pan H. Chun E.. (2019). The Crohn’s disease polymorphism, Atg16l1 T300a, alters the gut microbiota and enhances the local Th1/Th17 response. Elife 8:e39982. doi: 10.7554/ELIFE.39982 30666959 PMC6342529

[ref22] Law K. P. Lim Y. P. (2013). Recent advances in mass spectrometry: data independent analysis and hyper reaction monitoring. Expert Rev. Proteomics 10, 551–566. Epub 2013/11/12. doi: 10.1586/14789450.2013.858022, PMID: 24206228

[ref23] Lin J. C. Wu J. Q. Wang F. Tang F. Y. Sun J. Xu B.. (2019). Qingbai decoction regulates intestinal permeability of dextran sulphate sodium-induced colitis through the modulation of notch and Nf-Κb Signalling. Cell Prolif. 52:e12547. doi: 10.1111/cpr.12547, PMID: 30657238 PMC6496276

[ref24] Liu M. (2006). Modulation of immune function in rats with experimental ulcerative colitis by the abdominal massaging method. J. Chin. Med. 56+62. doi: 10.13463/j.cnki.jlzyy.2006.01.050

[ref25] Magoč T. Salzberg S. L. (2011). Flash: fast length adjustment of short reads to improve genome assemblies. Bioinformatics 27, 2957–2963. Epub 2011/09/10. doi: 10.1093/bioinformatics/btr507, PMID: 21903629 PMC3198573

[ref26] Mi H. Muruganujan A. Ebert D. Huang X. Thomas P. D. (2019). Panther version 14: more genomes, a new panther go-slim and improvements in enrichment analysis tools. Nucleic Acids Res. 47, D419–D426. Epub 2018/11/09. doi: 10.1093/nar/gky1038, PMID: 30407594 PMC6323939

[ref27] Mori H. Ohsawa H. Tanaka T. H. Taniwaki E. Leisman G. Nishijo K. (2004). Effect of massage on blood flow and muscle fatigue following isometric lumbar exercise. Med. Sci. Monit. 10, CR173–CR178. PMID: 15114265

[ref28] Mukhopadhya I. Hansen R. El-Omar E. M. Hold G. L. (2012). Ibd-what role do Proteobacteria play? Nat. Rev. Gastroenterol. Hepatol. 9, 219–230. doi: 10.1038/nrgastro.2012.14, PMID: 22349170

[ref29] Negahban H. Rezaie S. Goharpey S. (2013). Massage therapy and exercise therapy in patients with multiple sclerosis: a randomized controlled pilot study. Clin. Rehabil. 27, 1126–1136. Epub 2013/07/06. doi: 10.1177/0269215513491586, PMID: 23828184

[ref30] Ng S. C. Shi H. Y. Hamidi N. Underwood F. E. Tang W. Benchimol E. I.. (2017). Worldwide incidence and prevalence of inflammatory bowel disease in the 21st century: a systematic review of population-based studies. Lancet 390, 2769–2778. doi: 10.1016/S0140-6736(17)32448-0, PMID: 29050646

[ref31] Ni J. Wu G. D. Albenberg L. Tomov V. T. (2017). Gut microbiota and Ibd: causation or correlation? Nat. Rev. Gastroenterol. Hepatol. 14, 573–584. doi: 10.1038/nrgastro.2017.88, PMID: 28743984 PMC5880536

[ref32] Nitta M. Hirata I. Toshina K. Murano M. Maemura K. Hamamoto N.. (2002). Expression of the Ep4 prostaglandin E2 receptor subtype with rat dextran sodium Sulphate colitis: colitis suppression by a selective agonist, Ono-Ae1-329. Scand. J. Immunol. 56, 66–75. doi: 10.1046/J.1365-3083.2002.01096.X, PMID: 12100473

[ref33] Noah T. K. Shroyer N. F. (2013). Notch in the intestine: regulation of homeostasis and pathogenesis. Annu. Rev. Physiol. 75, 263–288. doi: 10.1146/annurev-physiol-030212-183741 23190077

[ref34] Owczarek D. Rodacki T. Domagała-Rodacka R. Cibor D. Mach T. (2016). Diet and nutritional factors in inflammatory bowel diseases. World J. Gastroenterol. 22, 895–905. doi: 10.3748/wjg.v22.i3.895, PMID: 26811635 PMC4716043

[ref35] Oxelmark L. Lindberg A. Löfberg R. Sternby B. Eriksson A. Almer S.. (2016). SOIBD, the Swedish organization for the study of inflammatory bowel disease use of complementary and alternative medicine in Swedish patients with inflammatory bowel disease: a controlled study. Eur. J. Gastroenterol. Hepatol. 28, 1320–1328. doi: 10.1097/MEG.0000000000000710, PMID: 27472271 PMC5051534

[ref36] Pope J. L. Bhat A. A. Sharma A. Ahmad R. Krishnan M. Washington M. K.. (2014). Claudin-1 regulates intestinal epithelial homeostasis through the modulation of notch-signalling. Gut 63, 622–634. doi: 10.1136/gutjnl-2012-304241, PMID: 23766441 PMC4083824

[ref37] Qi Q. Liu Y. N. Jin X. M. Zhang L. S. Wang C. Bao C. H.. (2018). Moxibustion treatment modulates the gut microbiota and immune function in a dextran sulphate sodium-induced colitis rat model. World J. Gastroenterol. 24, 3130–3144. doi: 10.3748/wjg.v24.i28.3130, PMID: 30065559 PMC6064969

[ref38] Qu L. Z. Zhao H. C. Shen H. X. Yan Y. Ma S. J. Wang K. Q. (2020). Electroacupuncture of both single- and multi-Acupoints promotes recovery of gastrointestinal function in laparoscopic cholecystectomy patients. Zhen Ci Yan Jiu 45, 136–140. doi: 10.13702/j.1000-0607.1904016, PMID: 32144924

[ref39] Rawsthorne P. Clara I. Graff L. A. Bernstein K. I. Carr R. Walker J. R.. (2012). The Manitoba inflammatory bowel disease cohort study: a prospective longitudinal evaluation of the use of complementary and alternative medicine services and products. Gut 61, 521–527. doi: 10.1136/gutjnl-2011-300219 21836028

[ref40] Schwab M. Klotz U. (2001). Pharmacokinetic considerations in the treatment of inflammatory bowel disease. Clin. Pharmacokinet. 40, 723–751. doi: 10.2165/00003088-200140100-00003, PMID: 11707060

[ref41] Skupsky J. Sabui S. Hwang M. Nakasaki M. Cahalan M. D. Said H. M. (2020). Biotin supplementation ameliorates murine colitis by preventing Nf-Κb activation. Cell Mol. Gastroenterol. Hepatol. 9, 557–567. doi: 10.1016/j.jcmgh.2019.11.011, PMID: 31786364 PMC7078531

[ref42] Sýkora J. Pomahačová R. Kreslová M. Cvalínová D. Štych P. Schwarz J. (2018). Current global trends in the incidence of pediatric-onset inflammatory bowel disease. World J. Gastroenterol. 24, 2741–2763. doi: 10.3748/wjg.v24.i25.2741, PMID: 29991879 PMC6034144

[ref43] Tefas C. Ciobanu L. Tanțău M. Moraru C. Socaciu C. (2020). The potential of metabolic and lipid profiling in inflammatory bowel diseases: a pilot study. Bosn. J. Basic Med. Sci. 20:262. doi: 10.17305/bjbms.2019.4235, PMID: 31368421 PMC7202185

[ref44] Tremaroli V. Bäckhed F. (2012). Functional interactions between the gut microbiota and host metabolism. Nature 489, 242–249. doi: 10.1038/nature11552 22972297

[ref45] Tsuboi K. Nishitani M. Takakura A. Imai Y. Komatsu M. Kawashima H. (2015). Autophagy protects against colitis by the maintenance of normal gut microflora and secretion of mucus. J. Biol. Chem. 290:20511. doi: 10.1074/jbc.M114.632257, PMID: 26149685 PMC4536456

[ref46] Ueda Y. Kawakami Y. Kunii D. Okada H. Azuma M. Le D. S. N. T.. (2008). Elevated concentrations of linoleic acid in erythrocyte membrane phospholipids in patients with inflammatory bowel disease. Nutr. Res. 28:239. doi: 10.1016/j.nutres.2008.02.005, PMID: 19083414

[ref47] Wang Q. Garrity G. M. Tiedje J. M. Cole J. R. (2007). Naive Bayesian classifier for rapid assignment of Rrna sequences into the new bacterial taxonomy. Appl. Environ. Microbiol. 73, 5261–5267. Epub 2007/06/26. doi: 10.1128/aem.00062-07, PMID: 17586664 PMC1950982

[ref48] Wang J. Liang Q. Zhao Q. Tang Q. Ahmed A. F. Zhang Y.. (2021). The effect of microbial composition and proteomic on improvement of functional constipation by chrysanthemum Morifolium polysaccharide. Food Chem. Toxicol. 153:112305. doi: 10.1016/j.fct.2021.112305 34033886

[ref49] Wang J. Wang X. Ma X. Xu B. Chen L. Chen C.. (2022). Therapeutic effect of Patrinia Villosa on Tnbs-induced ulcerative colitis via metabolism, vitamin D receptor and Nf-Κb signaling pathways. J. Ethnopharmacol. 288:114989. doi: 10.1016/J.JEP.2022.114989, PMID: 35032589

[ref50] Wang S. J. Yang H. Y. Wang F. Li S. T. (2015). Acupoint specificity on colorectal hypersensitivity alleviated by acupuncture and the correlation with the brain–gut Axis. Neurochem. Res. 40, 1274–1282. doi: 10.1007/s11064-015-1587-0 25968478

[ref51] Wei D. Xie L. Zhuang Z. Zhao N. Huang B. Tang Y.. (2019). Gut microbiota: a new strategy to study the mechanism of Electroacupuncture and Moxibustion in treating ulcerative colitis. Evid. Based Complement. Alternat. Med. 2019, 1–16. doi: 10.1155/2019/9730176, PMID: 31354859 PMC6632505

[ref52] Windsor J. W. Kaplan G. G. (2019). Evolving epidemiology of Ibd. Curr. Gastroenterol. Rep. 21:40. doi: 10.1007/s11894-019-0705-6, PMID: 31338613

[ref53] Wu H. Chen Q. Liu J. Chen X. Luo H. Ye Z.. (2021). Microbiome analysis reveals gut microbiota alteration in mice with the effect of matrine. Microb. Pathog. 156:104926. doi: 10.1016/j.micpath.2021.104926, PMID: 33964419

[ref54] Wu H. Chen Q. Y. Wang W. Z. Chu S. Liu X. X. Liu Y. J.. (2021). Compound Sophorae decoction enhances intestinal barrier function of dextran sodium sulfate induced colitis via regulating notch signaling pathway in mice. Biomed. Pharmacother. 133:110937. doi: 10.1016/j.biopha.2020.110937, PMID: 33217689

[ref55] Xiong T. Zheng X. Zhang K. Wu H. Dong Y. Zhou F.. (2022). Ganluyin ameliorates Dss-induced ulcerative colitis by inhibiting the enteric-origin Lps/Tlr4/Nf-Κb pathway. J. Ethnopharmacol. 289:115001. doi: 10.1016/J.JEP.2022.115001, PMID: 35085745

[ref56] Yang L. Liu C. Zhao W. He C. Ding J. Dai R.. (2018). Impaired autophagy in intestinal epithelial cells alters gut microbiota and host immune responses. Appl. Environ. Microbiol. 84:e00880-18. doi: 10.1128/AEM.00880-18, PMID: 30006408 PMC6121970

[ref57] Yu J. Zhi-gang L. Le-chun C. Huan-zhen C. J-c Z. H. A. N. G. (2019). Clinical observation of abdominal massage in treatment of ulcerative colitis. Chin. Manip. Rehabil. Med. 10, 28–30. doi: 10.19787/j.issn.1008-1879.2019.23.12

[ref58] Zeng H. Larson K. J. Cheng W. H. Bukowski M. R. Safratowich B. D. Liu Z.. (2020). Advanced liver Steatosis accompanies an increase in hepatic inflammation, colonic, secondary bile acids and Lactobacillaceae/Lachnospiraceae bacteria in C57bl/6 mice fed a high-fat diet. J. Nutr. Biochem. 78:108336. doi: 10.1016/j.jnutbio.2019.108336 32004929

[ref59] Zhu Y. Xiong Y. Gu Y. Li Q. Liu Y. (2020). Chiropractic therapy modulated gut microbiota and attenuated allergic airway inflammation in an immature rat model. Med. Sci. Monit. 26:e926039. doi: 10.12659/MSM.926039, PMID: 32990279 PMC7532697

